# Afghan Frontier: Understanding Tobacco Practices among Migrant Population in India

**DOI:** 10.31557/APJCP.2020.21.7.1931

**Published:** 2020-07

**Authors:** Rashmi Mehra, Vikrant Ranjan Mohanty, Aswini Y B, Karn Mehra, Shivam Kapoor

**Affiliations:** 1 *Department of Public Health Dentistry, Maulana Azad Institute of Dental Sciences, New Delhi, India. *; 2 *Department of Pulmonary Medicine Max Saket Super Speciality Hospital, New Delhi, India. *

**Keywords:** Tobacco use, refugees, tobacco cessation, migrants, India

## Abstract

**Background:**

According to the World Refugee Survey by US Committee for Refugees and Immigrants, migrants from Afghanistan constitute the 4th largest group of migrants to India. No previous study has been conducted to assess the tobacco consumption status and pattern among this marginalised migrant population.

**Aim:**

To get an insight of the tobacco usage pattern, accessibility and attitude towards tobacco cessation among the migrant population from Afghanistan.

**Methodology:**

A cross-sectional study was conducted among a convenience sample of Afghan tobacco users residing in Delhi, India in the month of June 2017. A close-ended self-administered validated questionnaire in Persian language was distributed at local cafés and restaurants.

**Statistical analysis:**

Data was entered in MS Excel Spreadsheet and descriptive statistics using SPSS version 21 were carried out.

**Results:**

A total of 127 male Afghan tobacco users with mean age of 33.49± 11.97 years completed the questionnaire. Better work opportunities were the most common reason for migration. Most of them (69%) smoked tobacco and 15.5% used only Naswar. Half (52%) of the respondents continue to use tobacco products manufactured in Afghanistan with 62% procuring the product through social means (friends/family). On assessing barriers to tobacco use, majority (85%) found higher cost of tobacco products to be a deterrent while19% agreed lack of availability to be a barrier. 50% felt that law enforcement and tobacco use regulation in India curbed their tobacco use. Tobacco usage was a stress buster for 64% of the respondents. Although 72% were interested in quitting tobacco, 58% feared losing friends if they quit. Strikingly, 93% were unaware about the availability of cessation services.

**Conclusion:**

Even as Afghans migrate from their homeland, they carry their cultural and social practices with them, including tobacco products, patterns and practices.

## Introduction

Culture is all-encompassing that permeates every aspect and level of human existence. The variation across cultures and ethnic groups in the prevalence and habits of tobacco use makes the importance of this understanding imperative. Tobacco is used throughout the world, but culture shapes the specific methods and patterns of its use.

There is substantial variation in tobacco use attributable to demographic factors, such as gender, socioeconomic status, age and migration history within broader racial ethnic or national groups (Global adult tobacco survey 2, 2017). Implicit in the twin studies analyses is the finding that a substantial proportion of the variance in smoking initiation and nicotine dependence is non-genetic, that is, attributable to environmental factors. Nearly all of the environmental factors known to be associated with tobacco use are directly or indirectly influenced by the cultural context (Heath, 1999; Banerjee et al., 2014). 

India is the third largest tobacco producing nation and second largest consumer of tobacco world-wide. Mortality due to tobacco in India is estimated at upwards of 1.3 million (Jha, 2008; Sinha, 2014). Global Adult Tobacco Survey (2017) revealed that 28.6 percent (266.8 million) of adults in India aged 15 and above currently use tobacco in some form. In urban areas, khaini (6.8%) and gutka (6.3%) are the two most commonly used tobacco products; whereas in rural areas khaini (13.5%) and bidi (9.3%) are the most prevalent tobacco products (GATS 2, 2017).

Owing to the growing tobacco epidemic, the Government of India began regulatory action towards tobacco control in 2003 with the enactment of the Cigarettes and Other Tobacco Products (Prohibition of Advertisement and Regulation of Trade and Commerce, Production, Supply and Distribution) Act, 2003 (COTPA). India has been one of the earliest nations to ratify the World Health Organization Framework Convention on Tobacco Control (WHO FCTC) in 2004. In 2007-08 India launched its National Tobacco Control Program. By legal provision smoking is completely banned in most public places and workplaces. All forms of tobacco advertising, promotion and sponsorship are prohibited. It is mandatory to have pictorial and text health warning labels on the tobacco product packages

According to the United Nations High Commissioner for Refugees (UNHCR), the second largest group of population of concern, who are not aided by the Government of India, hail from Afghanistan (UNHCR, 2018). While the South Asian Human Rights Documentation Centre (SAHRDC) estimates that approximately 60,000 Afghans live in India (South Asia Human Rights Documentation Centre, 1999). In terms of tobacco use, active cultural retention helps to reinforce traditional cultural ties and the norms regarding smoking and health. As ties to traditional culture weaken through the loss of ethnic culture, smoking patterns may change (Adlaf, 1989; Epstein, 1998; Bachman et al., 2011).

Limited data is available for the prevalence of tobacco use in Afghanistan (Bhatta et al., 2019). Recent surveys in Afghan cities estimated that 35.2% of men 15 years or older smoked with 85.4% had been at some time in their lives exposed to second-hand cigarette smoke (Khalil, 2003) and an overall prevalence of nass use was 48.8% (95% CI: 43.8–53.9%) (Humrah et al., 2018). The Global Youth Tobacco Survey (2010) found 90% of those who smoked started as teens, becoming smokers as early as 13 years old. Across the population, 82% of boys and 17% of girls have tried tobacco (GYTS 2006). The Global School-based Student Health Survey (GSHS) conducted in 2014, found that 7.9% of school children smoked cigarettes while 7.5% used some form of tobacco other than smoking (Shaikh, 2020).

Afghanistan had signed and ratified the WHO Framework Convention on Tobacco Control (WHO FCTC) in 2010. However, according to the 2017 WHO report on the global tobacco epidemic, Afghanistan is lagging in the implementation of most components of MPOWER in comparison to India. The most glaring aspect being the affordability and poor health warning implementation on tobacco products. A standardised packet of 20 cigarettes is 9 times more expensive in India in comparison to Afghanistan. At the same time, Afghanistan lacks a national campaign against tobacco and there is poor fund allocation enforcement of smoke free environment. With no national quit line, it falls short in providing its citizens in providing cessation services (WHO, 2018).

In India, due to better implementation of the WHO FCTC, there is heavy taxation, enforced health warnings and stricter regulation on tobacco use. This could possibly act as a deterrent or barrier to tobacco use among the Afghan migrants pushing them to resort to either cessation or other means to hold on to their tobacco practices while in India. It has been suggested that among determinants of smoking, most were variable according to ethnic group. There is a need for program planners and researchers should pay close attention to a number of factors when developing or examining smoking cessation or prevention programs for ethnically diverse groups. These factors include gender differences in smoking rates, the experience of immigration and its influence on smoking patterns, the social and cultural meaning of smoking. However, no studies have been conducted on this group of migrant population. Hence the aim of our study was to assess and get an insight about the current tobacco practices, attitudes and perceived barriers to tobacco use and cessation services among the Afghan migrants living in Delhi, India.

## Materials and Methods

*Methodology*

The present cross-sectional study was conducted among adult Afghan tobacco users (in restaurants and cafes), using a self-administered questionnaire. The study was conducted in 3 stages where the first stage involved developing a questionnaire to assess the practices and patterns of tobacco usage among Afghan migrants. A close ended questionnaire was developed, validated and translated in Persian language which is the vernacular for Afghan migrants. This survey underwent a pilot testing procedure with 15 Afghan tobacco users before the implementation of the final study to ensure validity (face) and reliability. As part of this, the surveys were translated (English to Persian) by a language expert and back-translated (Persian to English) for translation reliability. No significant differences in responses were noted between those surveyed in different languages. The questionnaire included questions on socioeconomic status, reason for migration, current and previous tobacco usage patterns and practices. It also included questions on quit attempts, barriers perceived to tobacco use and cessation in India. 

A list of all Afghan cafes/restaurants in Delhi was obtained from an extensive online search, totalling 46 cafes/restaurants. In the first stage, two cafes/restaurants were randomly selected for pilot survey procedures (obtaining the cooperation of restaurant owners, sampling of customers, and data collection procedures). A local Afghan community leader who had been residing in India for 15 years helped us penetrate into the sceptical and closed local Afghan community. To avoid selection bias, participants were not recruited from healthcare facilities or physician practices. Subsequently, in the five randomly selected cafes/restaurants, sampling of customers was done by randomly selecting the ones to be interviewed. Number of tobacco consuming Afghan migrants interviewed from each cafe/restaurant depended on time mainly (the research team spent, on average, 4 h in each location in order not to interfere much with the usual work at cafes/restaurants). Usually customers sat in groups and spent about 1–2 hours while eating, drinking, and talking.

Only those adult Afghans who consumed tobacco, had been residing in India for at least 6 months, who could read Persian language and were willing to participate in the study were given the self-administered questionnaire. The protocol for the study and informed consent documents were approved by the Institutional Review Boards at Maulana Azad Institute of Dental Sciences. The data obtained from completed questionnaires was entered onto MS Excel spreadsheet. Mean and standard deviation values were derived for continuous variables. Descriptive inferences were drawn for nominal data.

## Results

A total of 127 participants willingly completed the self-administered questionnaire and hence were included in the study across the 5 randomly selected Afghan Cafes and restaurants. All participants of the study were male with mean age of 33.14±11.59 years. Most had completed graduation and were unemployed at the moment in India. Many (48.4%) of the participants had completed graduation with 28.6% pursuing regular jobs. Most of the participants (45.6%) had been residing in India for the last 3 years or more and 79.4% were married. About 40% of the participants migrated to India in search for better work opportunities, with unemployment among participants plummeting down from 38% in Afghanistan to 15.1% in India among the migrant community. The demographic features of the study population are enlisted in Table 1.

**Figure 1.**
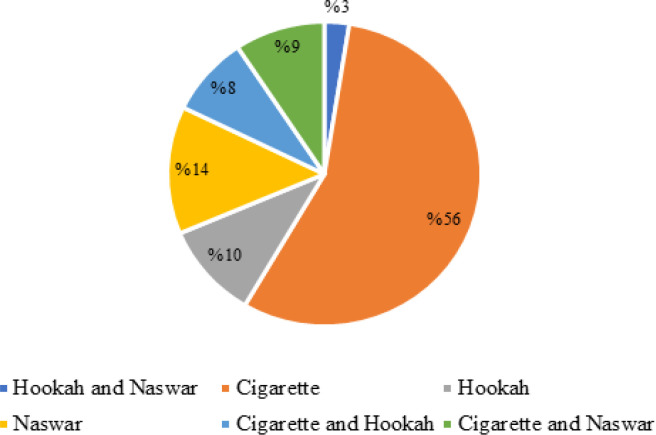
Types of Tobacco Products Used by Afghan Migrants in India

Majority of the participants used smoked form of tobacco (70.4%) with 55.9% using cigarettes followed by 13.6% using Naswar (a form of smokeless tobacco popularly used in Afghanistan) as shown in Figure 1. Factors such as peer group (53.3%), stress (36.7%) and staying away from family (6.7%) had reportedly resulted in 21.6% of the participants initiating tobacco use in India.

Another interesting finding was that 89% of participants used tobacco products which were not manufactured in India and 44.9% used tobacco products manufactured in Afghanistan. 

**Figure 2:**
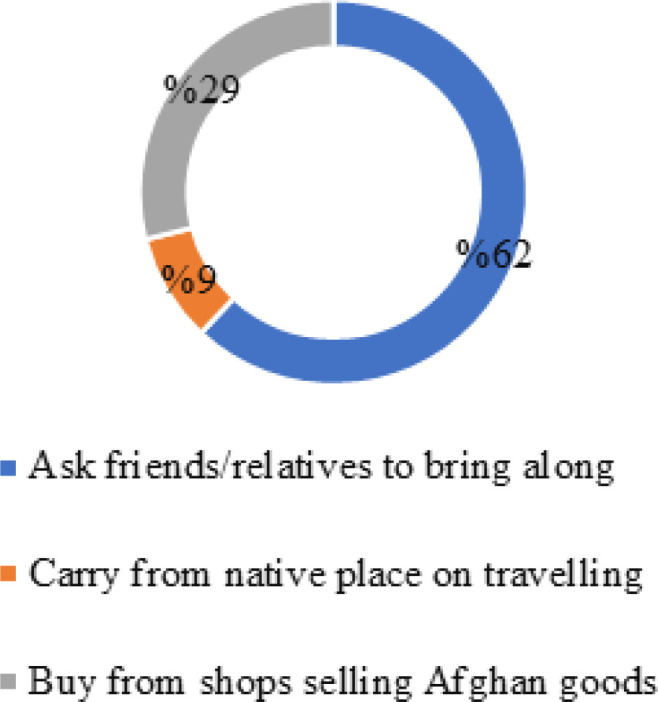
Types of Tobacco Products Used by Afghan Migrants in India

As depicted in Figure 2, about 44.8% of study participants reported a difference in the tobacco product they were previously using in their homeland to the one they currently used with most (66.7%) reporting native tobacco product to be “better”. This culminated in many resorting to procuring the native tobacco products through friends and family who were travelling (62%) or carried the product in bulk from Afghanistan (28.9%). Others resorted to buying the tobacco product of their choice from a store which sells Afghan and imported products. 

When assessed for the barriers faced for tobacco use in India most found India to be restrictive to their tobacco use habit and pattern. The results for the same have been represented in Table 2 followed by the barriers faced to quitting tobacco in Table 3. The mean number of quit attempts were 1.85 ±2.737. The seemingly biggest barrier to tobacco use among Afghan migrants was found to be the cost of the products in India followed by limited or lack of availability of the product of their choice in the Indian market. There was equivocal strength of law enforcements being a barrier to tobacco use. Paucity of social involvement which usually was associated with tobacco use in their home land was also cited as a barrier to tobacco use.

On assessing the barriers to quitting tobacco use it was found that the fear of losing friends on quitting tobacco was expressed by 58.2% of the participants. Almost all participants (92%) did not know where to access help to quit their tobacco consumption habit. Interestingly, 75% participants were not interested in quitting even though only 23% admitted to be unaware of the ill effects caused by tobacco use. Many also voiced that the lack of tobacco cessation services was a barrier to quitting the habit.

**Table 1:** The Demographic Features of The Study Population

Demographic Features	Percentage (%)	Frequency (n)
Gender		
Male	100	127
Female	0	0
Marital Status		
Married	79.4	101
Single	20.6	26
Divorced/Widowed	0	0
Education Level		
Primary/Middle School	10.3	13
High School	29.4	37
Graduation	48.4	62
Post-Graduation	11.9	15
Current Occupation		
Unemployed	38.1	49
Job	28.6	36
Business	33.3	42
Previous Occupation in Afghanistan		
Unemployed	15.1	19
Job	47.6	61
Business	37.3	47

**Table 2:** Barriers for Tobacco Use in India among Migrant Afghan Tobacco Users

Barriers for tobacco use in India	Yes	No	Can't say
Lack of Availability of tobacco product of your native place is a barrier to tobacco use:	24	100	3
Cost of tobacco product of in India is a barrier to tobacco use	108	8	11
Law enforcement and tobacco use regulation in India is a barrier to tobacco use	63	63	1
Paucity of social involvement is a barrier to tobacco use	73	52	2

**Figure 3.**
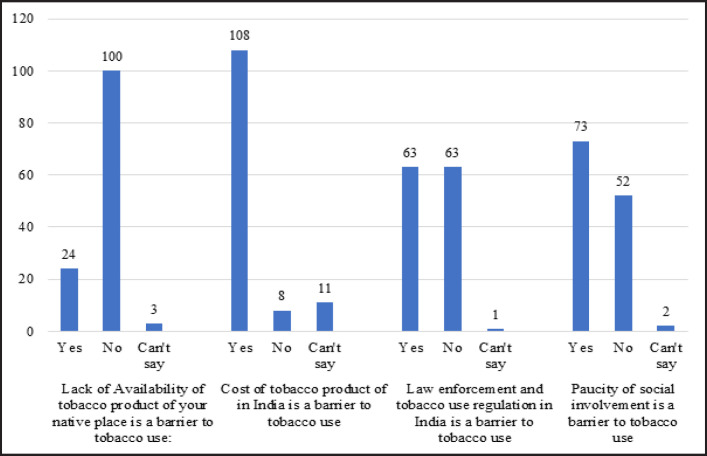
Barriers to Tobacco Use

**Figure 4:**
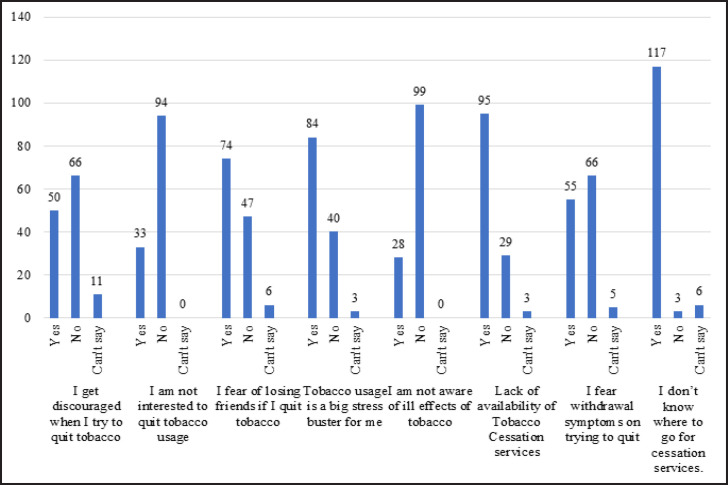
Barriers to Quit Tobacco Use

**Table 3:** Barriers to Quitting Tobacco among Migrant Afghan Tobacco Users

Barriers to quitting tobacco	Yes	No	Can’t Say
I get discouraged when I try to quit tobacco	50	66	11
I am not interested to quit tobacco usage	33	94	0
I fear of losing friends if I quit tobacco	74	47	6
Tobacco usage is a big stress buster for me	84	40	3
I am not aware of ill effects of tobacco	28	99	0
Lack of availability of Tobacco Cessation services	95	29	3
I fear withdrawal symptoms on trying to quit	55	66	5
I don’t know where to go for cessation services.	117	3	6

## Discussion

Human Migration is the movement of people from their home turf to a land which they hope to call their own. In the quest for a better life, many are uprooted from their homes where they were probably denied the essential freedoms of life and move elsewhere for survival and a life with dignity. They are international refugees and migrants, fleeing from their country, where they fear or have suffered oppression. Several military, social and political conflicts have brought in their wake an innumerable number of uprooted, including millions of refugees in search of new homes. Many such homes were provided by many neighbouring countries of the war struck nations, India being one of them. Migrants from Afghanistan constitute a major portion of migrants living in India. 

It has been seen that the response of different peoples to the experience of dislocation varies tremendously as does the ability of different social groups to maintain their cultural identity and their traditional social structure and institutions (De Voe DM, 1981; Jayawickreme and Blackie, 2016; Shin, 2017). Social structure and prevailing societal conditions have long been implicated in shaping individual health behaviours including tobacco use practices. Numerous factors culminate into an individual tobacco use practice and pattern including the socio-economic and political policies and situation. While material, social capital, psychosocial factors and prevailing level of health care initiatives affect tobacco use proximally (Bernabé, 2012).

As seen in our study majority of the Afghan tobacco users continued to use the tobacco product they used in their native country, which are not manufactured in India. This puts forth the pertinent question of procuring foreign products by circumventing the laws and regulations under the Cigarette and Other Tobacco Products Act 2003 and articles of FCTC. Such practices act as a threat to tobacco control measures. It would only be understandable that such a phenomenon will not be limited to India but poses as a threat to other countries too. According to the migration profile of Afghanistan, a total of 48,55,068 migrants moved from Afghanistan mostly to Pakistan, Iran, Germany, United Kingdom and United States of America (United Nations Database, 2013). As seen in our study, the regulation on tobacco use acts as a barrier for the migrant group due to higher costs and poorer product availability which are results of stricter implementation of the COTPA and FCTC.

Among our study participants, most participants reported smoking with other fellow Afghans, however our study could not assess the exposure to second hand smoke among this community which owing to the group behaviour of tobacco consumption must be alarming. Since the female populace, is stricken by prejudice and conservatism, it was not possible for us to divulge tobacco use pattern among the women folk of this community. A greater involvement and support of local female leader might prove useful to do the same and penetrate into the community. 

Another impending issue brought forth by our study is about the lack of awareness about tobacco cessation services among this group even when most wanted to quit the habit. Majority of the participants did not fear the withdrawal symptoms they might face upon quitting, instead feared losing out on their friends if they quit. This finding is suggestive of the enormous impact the socio-cultural influences have on the initiation and continuation of tobacco use which is in line with context presented by Unger (2003).

We believe that our recent observations provide a compelling argument for the development of smoking cessation programmes that are tailored to the differences in addictive behaviour and counselling requirements of smokers from various cultural backgrounds. The important features of such cessation programmes would include offering individual or group counselling in the participants’ native language, providing informative material and counselling in schools and mosques, recruitment via media in the respective mother tongue, predominantly visual and oral information, education about smoke-related diseases, education about nicotine replacement products and avoidance strategies, as well as regular follow-up visits to evaluate treatment progress. The limitations of our study include the fact that participants were recruited in public places, which constitutes a potential source of selection bias.

Moreover, the results might not directly be generalisable to other countries as the study was conducted exclusively in Delhi. The cross-sectional study design represents a limitation of our data, as a longitudinal assessment would have provided further insight into trends of tobacco use. However, we aimed at prompt availability of the data to claim rapid implementation of corresponding public health interventions.

In conclusion, it was found that most Afghan tobacco users used smoked form of tobacco, primarily cigarettes. The increased cost of tobacco products in India was a major barrier to tobacco use for this migrant group. Even though majority expressed that they would like to quit the habit, most of them were unaware of existing tobacco cessation services. The Afghan tobacco users continue using products they used in their homeland as it probably gives them a sense of culture retention and homeliness in a land that is unknown to their culture, their ethos and their customs. 

## References

[B1] Adlaf EM, Smart RG, Tan SH (1989). Ethnicity and drug use: A critical look. Int J Addict.

[B2] Bachman JG, O’Malley PM, Johnston LD, Schulenberg JE, Wallace Jr JM (2011). Racial/ethnic differences in the relationship between parental education and substance use among US 8th-, 10th-, and 12th-grade students: findings from the Monitoring the Future project. J Stud Alcohol Drug.

[B3] Bhatta DN, Hiatt RA, Van Loon K, Glantz SA. (2019). Exposure to household tobacco smoke and risk of cancer morbidity and mortality: Analysis of data from the Afghanistan Demographic and Health Survey 2015. Prev Med.

[B4] Banerjee SC, Ostroff JS, D’Agostino TA (2014). Disengagement beliefs in South Asian immigrant smokeless tobacco users: A qualitative study. Addict Res Theory.

[B5] Beardall S, Edwards N (1995). Social and cultural determinants of smoking behavior in selected immigrant groups: Results of key informant interviews. Fam Community Health.

[B6] Bernabé E, Watt RG, Sheiham A (2012). Childhood socioeconomic position, adult sense of coherence and tooth retention. Community Dent Oral Epidemiol.

[B7] Bush J, White M, Kai J, Rankin J, Bhopal R (2003). Understanding influences on smoking in Bangladeshi and Pakistani adults: community based, qualitative study. Br Med J.

[B8] Banerjee SC, Ostroff JS, Bari S (2014). Gutka and Tambaku Paan use among South Asian immigrants: a focus group study. J Immigr Minor Health.

[B10] De Voe DM Framing refugees as clients. Int Migrat Rev.

[B11] Epstein JA, Botvin GJ, Diaz T (1998). Ethnic and gender differences in smoking prevalence among a longitudinal sample of inner-city adolescents. J Adolesc Health.

[B12] Gavlak D (2008). Smoke alarm from Afghanistan to Morocco. Bulletin of the World Health Organization.

[B14] Hamrah MH, Hamrah MS, Hamrah MH (2018). Nass use and associated factors among outpatients in northern Afghanistan: A cross-sectional study in Andkhoy City. Tob Induc Dis.

[B15] Heath AC, Kirk KM, Meyer JM, Martin NG 1999), Genetic and social determinants of initiation and age at onset of smoking in Australian twins. Behav Genet.

[B18] Jha P, Jacob B, Gajalakshmi V (2008). A nationally representative case–control study of smoking and death in India. New Eng J Med.

[B22] Nápoles-Springer A, Pérez-Stable EJ (2001). The role of culture and language in determining best practices. J Gen Intern Med.

[B23] Shaikh MA (2020). Tobacco use in school students in Afghanistan, Oman and Kuwait and association with parental monitoring: analysis of data from Global School-based Student Health surveys. East Mediterranean Health J.

[B25] Sinha DN, Palipudi KM, Gupta PC et al (2014). Smokeless tobacco use: a meta-analysis of risk and attributable mortality estimates for India. Indian J Cancer.

[B29] Urban M, Burghuber OC, Dereci C (2015). Tobacco addiction and smoking cessation in Austrian migrants: a cross-sectional study. Br Med J Open.

[B32] Unger JB, Cruz T, Baezconde-Garbanati L (2003). Exploring the cultural context of tobacco use: a transdisciplinary framework. Nicotine Tob Res.

[B33] Wann KD (1959). Cultural-values and learning in Afghanistan. Educ Leadership.

